# Acute Pain Management in Children: A Systematic Review and GRADE-Based Recommendations

**DOI:** 10.3390/jcm15155802

**Published:** 2026-07-24

**Authors:** Gregorio Paolo Milani, Matteo Riccò, Massimo Agosti, Rino Agostiniani, Angela Amigoni, Carla Aromatario, Egidio Barbi, Franca Benini, Antonella Cersosimo, Lucia De Zen, Daniela Dibello, Elena Chiappini, Antuan Divisic, Paola Frati, Dario Galante, Pablo Mauricio Ingelmo, Svetlana Kotzeva, Nicola Luxardo, Luca Manfredini, Carmelo Minardi, Luca Moresco, Anna Maria Moretti, Lorenzo Moscaritolo, Moreno Crotti Partel, Federico Pellegatta, Sofia Pitino, Barbara Rebesco, Emanuela Omodeo Salè, Patrizia Savant Levet, Rita Signorini, Alessandro Simonini, Marta Somaini, Agnese Suppiej, Stefano Sotgiu, Assunta Tornesello, Fabio Verdoni, Alessandro Vittori, Tiziana Zangardi, Simone Pilloni, Chiara Sassetti, Dragos Septelici, Giulia Brigadoi, Daniele Donà, Paola Lago, Giuseppe Squazzini, Susanna Esposito

**Affiliations:** 1 Società Italiana di Medicina di Emergenza e Urgenza Pediatrica (SIMEUP), Department of Pharmacy, Health and Nutritional Sciences, University of Calabria, Rende—Pediatric Unit, Annunziata Hospital, 87100 Cosenza, Italy; milani.gregoriop@gmail.com; 2 Società Italiana di Igiene (SITI), Unità Operativa Prevenzione e Sicurezza Ambienti di Lavoro, AUSL Reggio Emilia, 42122 Reggio Emilia, Italy; mricco2000@gmail.com; 3 Società Italiana di Neonatologia (SIN), Unità di Neonatologia Terapia Intensiva Neonatale e Pediatria, Università degli Studi dell’Insubria, 21100 Varese, Italy; massimo.agosti@uninsubria.it; 4 Department of Pediatrics and Neonatology, San Jacopo Hospital, Via Ciliegiole 97, 51100 Pistoia, Italy; rinoagostiniani@gmail.com; 5 Società di Anestesia e Rianimazione Neonatale e Pediatrica Italiana (SARNEPI), UOSD Terapia Intensiva Pediatrica, Azienda Ospedale-Università, 35128 Padova, Italy; angela.amigoni@aopd.veneto.it; 6 Società Italiana di Anestesia, Analgesia e Terapia Intensiva Pediatrica (SIAATIP), S.C. di Anestesia e Rianimazione, Ospedale Santo Spirito, 65124 Pescara, Italy; aromatariocarla@yahoo.it; 7 Società Italiana di Pediatria, Pediatric Department, Institute for Maternal and Child Health IRCCS “Burlo Garofolo”, 34137 Trieste, Italy; egidio.barbi@burlo.trieste.it; 8 Società Italiana di Cure Palliative (SICP), UOC Hospice Pediatrico, Azienda Ospedale-Università, 35128 Padova, Italy; franca.benini@aopd.veneto.it (F.B.); antuan.divisic@aopd.veneto.it (A.D.); 9 Società Italiana di Medicina Fisica e Riabilitativa (SIMFER), UOC Medicina Riabilitativa Infantile, IRCCS Istituto delle Scienze Neurologiche, 40124 Bologna, Italy; a.cersosimo@isnb.it; 10 Associazione Italia di Oncoematologia Pediatrica (AIEOP), Centro Regionale Terapia del Dolore e Cure Palliative Pediatriche, Istituto IRCCS “Burlo Garofolo”, 34137 Trieste, Italy; lucia.dezen@burlo.trieste.it; 11 Società Italiana di Ortopedia e Traumatologia Pediatrica (SITOP), UO Ortopedia e Traumatologia, Ospedale Pediatrico Giovanni XXIII, 70126 Bari, Italy; daniela.dibello@policlinico.ba.it; 12 Società Italiana di Pediatria Preventiva e Sociale (SIPPS), Ospedale Pediatrico Meyer IRCCS, Università di Firenze, 50139 Firenze, Italy; elena.chiappini@unifi.it; 13 Società Italiana di Medicina Legale e delle Assicurazioni (SIMLA), Sapienza Università di Roma, 00185 Roma, Italy; paola.frati@uniroma1.it; 14 Società Italiana di Anestesia, Analgesia e Terapia Intensiva Pediatrica (SIAATIP), S.C. Anestesia e Rianimazione, Azienda Ospedaliero Universitaria, Ospedali Riuniti di Foggia, 71122 Foggia, Italy; galante.dario@gmail.com; 15 Società di Anestesia e Rianimazione Neonatale e Pediatrica Italiana (SARNEPI), SSD Terapia del Dolore e Medicina Palliativa Pediatrica, Fondazione IRCCS San Gerardo dei Tintori di Monza, Università degli Studi di Milano-Bicocca, 20126 Monza, Italy; pablomauricio.ingelmo@unimib.it; 16 Società di Anestesia e Rianimazione Neonatale e Pediatrica Italiana (SARNEPI), UOC Anestesia e Terapia del Dolore Acuto e Procedurale, IRCCS Istituto Giannina Gaslini, 16147 Genova, Italy; svetlanakotzeva@gaslini.org; 17 Associazione Italiana per lo Studio del Dolore (AISD), S.C. Terapia del Dolore (Terapia Antalgica), ASL Città di Torino, 10128 Torino, Italy; nicolaluxardo58@gmail.com; 18 Società Italiana Medici Pediatri (SIMPE), Responsabile Hospice, UOSD—Ospedale Domiciliare, IRCCS Istituto Giannina Gaslini, 16147 Genova, Italy; lucamanfredini@gaslini.org; 19 Società di Anestesia e Rianimazione Neonatale e Pediatrica Italiana (SARNEPI), UOC Anestesia e Rianimazione, AOU Policlinico “G. Rodolico—San Marco”, 95123 Catania, Italy; minardi.carmelo@hotmail.com (C.M.); sofp85@gmail.com (S.P.); 20 Società Italiana Medici Pediatri (SIMPE), Referente Regionale FIMP per le Cure Palliative, ASL2 Liguria, 17100 Savona, Italy; morescol@inwind.it; 21 Società Scientifica di Medicina di Genere (GISEG), Santa Maria Hospital, 70124 Bari, Italy; moretti.am@libero.it; 22 Associazione Italiana per lo Studio del Dolore (AISD), Centro Interdisciplinare di Diagnosi e Cura del Dolore Complesso in Età Pediatrica, Ospedale Infantile Regina Margherita, 10126 Torino, Italy; lorenzo.moscaritolo@gmail.com; 23 Società Italiana di Pediatria Infermieristica (SIPINF), ASST Spedali Civili, 25123 Brescia, Italy; moreno.crottipartel@gmail.com; 24 Società Italiana di Pediatria Infermieristica (SIPINF), VIDAS ODV, 20151 Milano, Italy; federico.pellegatta@vidas.it; 25 Società Italiana di Farmacia Ospedaliera (SIFO), Politiche del Farmaco Regione Liguria, 16121 Genova, Italy; barbara.rebesco@gmail.com; 26 Società Italiana di Farmacia Ospedaliera (SIFO), Division of Pharmacy, European Institute of Oncology, IRCCS, 20141 Milan, Italy; eomodeo@ieo.it; 27 Società Italiana di Neonatologia (SIN), SC Terapia Intensiva Neonatale e Neonatologia, Ospedale Maria Vittoria, 10144 Torino, Italy; patrizia.savantlevet@aslcittaditorino.it; 28 Società Italiana delle Cure (SICuPP), Pediatra di Libera Scelta, 32100 Belluno, Italy; rit.sign@virgilio.it; 29 Società Italiana Anestesia Rianimazione e Terapia Intensiva (SIAARTI), SOD Anestesia e Rianimazione Pediatrica, Ospedale dei Bambini Salesi, 60126 Ancona, Italy; dr.simonini@gmail.com; 30 Società di Anestesia e Rianimazione Neonatale e Pediatrica Italiana (SARNEPI), ASST Grande Ospedale Metropolitano Niguarda, 20162 Milano, Italy; ma.somaini@gmail.com; 31 Società Italiana di Neurologia Pediatrica (SINP), UO Pediatria, Arcispedale Sant’Anna, Università degli Studi di Ferrara, 44124 Ferrara, Italy; agnese.suppiej@unife.it; 32 Società Italiana di Neuropsichiatria dell’Infanzia e dell’Adolescenza (SINPIA), Divisione di Neuropsichiatria Infantile, Azienda Ospedaliero Universitaria di Sassari, 07100 Sassari, Italy; stefanos@uniss.it; 33 Società Italiana Medici Pediatri (SIMPE), UO Oncoematologia Pediatrica, PO Vito Fazzi, 73100 Lecce, Italy; assuntatornesello@gmail.com; 34 Società Italiana di Ortopedia e Traumatologia Pediatrica (SITOP), UO Ortopedia Pediatrica, IRCCS Ospedale Galeazzi—Sant’Ambrogio, 20157 Milano, Italy; verdonifabio@alice.it; 35 Società Italiana Anestesia Rianimazione e Terapia Intensiva (SIAARTI), Ospedale Pediatrico Bambino Gesù IRCCS, 00165 Roma, Italy; alexvittori82@gmail.com; 36 Società Italiana di Medicina di Emergenza e Urgenza Pediatrica (SIMEUP), UOC Pronto Soccorso Pediatrico, Azienda Ospedale-Università, 35128 Padova, Italy; tiziana.zangardi@aopd.veneto.it; 37 Società Italiana di Telemedicina (SIT), UOC Clinica Pediatrica, Università di Parma, 43126 Parma, Italy; simone.pilloni@unipr.it (S.P.); chiara.sassetti@unipr.it (C.S.); dragos.septelici@unipr.it (D.S.); 38 Società Italiana di Pediatria, UOSD Malattie Infettive Pediatriche, Azienda Ospedale-Università, 35128 Padova, Italy; giulia.brigadoi@gmail.com (G.B.); daniele.dona@unipd.it (D.D.); 39 Società Italiana di Pediatria (SIP), UOC Terapia Intensiva Neonatale, Ospedale Cà Fondello, 31100 Treviso, Italy; paolalago9@gmail.com; 40 Società Italiana Medici Pediatri (SIMPE), Pediatra di Famiglia, 17027 Savona, Italy; vicepresidenza@simpeservizi.it

**Keywords:** pediatric pain, acute pain, pain assessment, analgesia, ibuprofen, non-pharmacological interventions

## Abstract

**Background**: Acute pain in children is frequently under-recognized and undertreated despite validated assessment tools and effective therapies. This systematic review aimed to synthesize recent evidence on assessment and management of acute pediatric pain. **Methods**: The review followed PRISMA 2020 guidelines. Clinical questions were developed using the PICO framework, and evidence certainty was assessed with GRADE. PubMed and Embase were searched for studies published from 2016 to 2025. Italian pediatric scientific societies and specialists involved in acute pediatric care contributed to the process. **Results**: A total of 13 studies were included. Validated pain scales showed high reliability and were associated with reduced pain scores after analgesic administration. Paracetamol and ibuprofen showed comparable efficacy for mild-to-moderate pain; evidence suggesting a modest late advantage of ibuprofen was inconsistent. Combination therapy reduced rescue analgesia in individual studies. Non-pharmacological interventions, including virtual reality, distraction, and hospital play, reduced anxiety and procedural distress, although certainty of evidence was low. Evidence on opioids was limited and did not show clear superiority over non-opioid strategies. Intranasal fentanyl appeared comparable to intravenous morphine and more feasible in emergency settings. Overall, evidence for several clinical questions remains limited, and GRADE recommendations were only possible for selected PICOs. **Conclusions**: Acute pediatric pain management should rely on structured assessment and multimodal strategies. Validated pain scales should be used routinely. Paracetamol and ibuprofen remain first-line treatments for mild-to-moderate pain, with possible context-specific benefits of ibuprofen or combination therapy. Non-pharmacological interventions may be useful adjuncts, especially for anxiety and procedural distress. Further high-quality studies are needed on opioids, intranasal administration, alternating regimens, and inhaled versus topical analgesia.

## 1. Introduction

Acute pain in children represents a critical component of clinical care, with significant implications for both short- and long-term outcomes. Inadequate pain management has been associated with increased procedural anxiety, behavioral changes, and long-lasting neurobiological alterations [1,2,3]. It is now well established that inadequate pain control in early life may negatively influence the healthcare experience, increase procedural anxiety, and contribute to persistent behavioral and neurobiological alterations [4,5,6].

Despite the wide availability of validated assessment tools and therapeutic options, the literature highlights significant heterogeneity in approaches to pediatric pain, with frequent delays in assessment, underestimation of pain intensity, and suboptimal use of analgesic strategies [3,7,8]. Observational studies conducted across various clinical settings report that pain in children is still frequently under-recognized or inadequately treated, particularly in emergency and inpatient settings [9,10,11], but also in out-of-hospital contexts [12,13]. Moreover, although previous systematic reviews have addressed specific aspects of pediatric pain management, these reviews have generally focused on single interventions or specific clinical settings. To our knowledge, no recent review has integrated the available evidence across the principal domains of acute pediatric pain management using predefined PICO questions and the GRADE framework to support clinically oriented recommendations.

This gap between recommendations and everyday clinical practice underscores the need for a systematic synthesis of the available evidence and for a structured framework to support the development of standardized practices. In response to this need, the present study aims to provide a systematic analysis of the literature on the management of acute pain in children and adolescents, structured around clinical questions formulated according to the PICO model. The objective is to critically integrate existing knowledge, identifying areas of evidence while also identifying the gaps that persist in pediatric acute pain research.

## 2. Materials and Methods

### 2.1. Study Design

This systematic review was conducted in accordance with the PRISMA 2020 (Supplementary Material S4, Preferred Reporting Items for Systematic Reviews and Meta-Analyses) guidelines. The review process was based on structured clinical questions developed using the PICO framework and followed a predefined methodological approach.

### 2.2. Development of Clinical Questions (Population, Intervention, Comparison, Outcome [PICO])

Clinical questions were developed through a structured process involving a multidisciplinary panel of experts, including pediatricians, anesthesiologists, emergency physicians, and pain specialists. An initial set of research questions was proposed and subsequently refined through iterative discussion among panel members. The final PICO questions were selected according to clinical relevance, frequency in routine pediatric practice, variability or uncertainty in current management, potential impact on patient outcomes, and feasibility of translation into evidence-based recommendations. A total of seven PICO questions were defined and prioritized based on clinical relevance (the full list is reported in the online Supplementary Material). Subsequent analyses were conducted separately for each PICO question.

### 2.3. Search Strategy

A comprehensive literature search was performed in PubMed and Embase databases to identify studies published up to December 2025. The search was intentionally restricted to studies published in this timeframe to provide recommendations based on the most contemporary evidence. This period reflects current clinical practice, incorporates recent advances in pain assessment tools, non-pharmacological interventions, and evolving analgesic strategies. Search strategies combined controlled vocabulary (MeSH and Emtree terms) and free-text keywords related to pediatric acute pain, pain assessment, and treatment strategies. The full search strategies for each PICO question are reported in the Supplementary Material S1. Screening was conducted independently by two reviewers using Rayyan software (Cambridge, MA, USA). Disagreements were resolved through discussion and consensus, and when necessary, by consultation with a third reviewer.

### 2.4. Eligibility Criteria

#### 2.4.1. Inclusion Criteria

Studies were included if they met the following criteria: pediatric population (1 month–18 years), acute pain conditions (procedural, traumatic, infectious, or postoperative), observational or interventional study design, original data with at least 10 participants, published in English, full-text availability, pertinence to at least one of the PICO.

#### 2.4.2. Exclusion Criteria

Studies were excluded if they involved neonates (<1 month), addressed chronic pain conditions, included mixed adult/pediatric populations without separate pediatric data, were reviews, editorials, case reports, or conference abstracts, or lacked sufficient methodological detail.

#### 2.4.3. Screening Process

Screening was conducted using Rayyan software.

### 2.5. Data Extraction

Data extraction was performed independently by two reviewers using a standardized, pre-defined data extraction form developed specifically for this review. For each included study, the following data were collected: (1) study characteristics (author, year, country, design, setting), (2) population characteristics (age, sample size, inclusion/exclusion criteria), (3) intervention and comparator details, (4) outcomes assessed, (5) main results. Any discrepancies between reviewers were resolved through consensus discussion.

### 2.6. Risk of Bias Assessment

The methodological quality of included studies was assessed independently by two reviewers.

- Randomized controlled trials (RCTs): Evaluated using the Cochrane Risk of Bias tool (RoB 2);
- Observational studies: Assessed using the Newcastle-Ottawa Scale.

Disagreements were resolved through discussion and consensus. If necessary, a third reviewer was involved to determine study eligibility.

### 2.7. Data Synthesis

Given the heterogeneity in study design, populations, and outcome measures, a qualitative synthesis of the evidence was performed. Quantitative synthesis (meta-analysis) was conducted only when studies were sufficiently homogeneous in terms of population, intervention, and outcomes. When meta-analysis was not feasible (in the absence of at least three comparable studies), results were summarized narratively. Cohen kappa was evaluated to assess reviewers’ agreement in study selection.

### 2.8. Certainty of Evidence (GRADE), Clinical Recommendations and Guidance

The certainty of evidence for each outcome was assessed using the GRADE (Grading of Recommendations Assessment, Development and Evaluation) approach. The following domains were considered: a. risk of bias, b. inconsistency, c. indirectness, d. imprecision, e. publication bias. The certainty of evidence was categorized as high, moderate, low, and very low. Formal GRADE assessment and evidence-based recommendations were developed only for PICO questions supported by sufficient evidence to allow a structured assessment of the certainty of evidence. In the present review, this corresponded to PICO questions for which at least three eligible studies were identified. When fewer than three studies were available, the evidence was considered insufficient to support formal GRADE assessment; in these cases, evidence-informed clinical guidance statements were formulated based on the available evidence and expert interpretation, rather than formal evidence-based recommendations.

#### Expert Consensus Process and Development of Recommendations

Following completion of the systematic review and, where applicable, the GRADE assessment, two investigators with expertise in evidence synthesis and guideline methodology independently drafted evidence-based clinical recommendations or evidence-informed clinical guidance statements for each PICO question. Formal recommendations were developed only for PICO questions supported by sufficient evidence to allow formal GRADE assessment, whereas evidence-informed clinical guidance statements were formulated for PICOs with limited or insufficient evidence. For these PICOs, expert opinion was used to support the interpretation and clinical contextualization of the available evidence rather than to replace it. Accordingly, these statements should be interpreted as evidence-informed clinical guidance rather than formal evidence-based recommendations. The draft recommendations and guidance statements were reviewed by a panel of five independent international experts in pediatric pain management, who evaluated their scientific accuracy, clinical relevance, clarity, feasibility, and consistency with the available evidence. The experts were also invited to identify additional relevant publications that had not been retrieved through the predefined search strategy whenever these were considered important for contextualizing the evidence, supporting interpretation of the findings, or informing the final statements. These references were considered complementary background evidence and did not modify the predefined eligibility criteria or the studies included in the systematic review. The revised recommendations and guidance statements were subsequently circulated to the representatives of all participating Italian scientific societies involved in pediatric pain management for review and approval before manuscript finalization. A formal Delphi methodology was not used. Instead, consensus was achieved through an iterative review process involving the expert panel and the representatives of the participating scientific societies. Potential bias was minimized through independent expert review followed by multidisciplinary evaluation and approval by the participating scientific societies. Whenever disagreements regarding the wording or content of a statement emerged, they were resolved through discussion until unanimous agreement was reached. Patients or external stakeholders were not involved in the consensus process.

## 3. Results

### 3.1. PICOs Identified

A total of seven PICO questions were developed through the expert panel process, addressing key domains of pediatric acute pain management, including pain assessment, pharmacological treatment, and non-pharmacological interventions. The PICO questions were defined as follows.

PICO 1—Pain assessment

In children with acute pain, does the use of validated pain assessment scales improve the accuracy of pain recognition and the adequacy of analgesic management compared to unstructured clinical assessment?

PICO 2—Paracetamol vs. ibuprofen

In children with mild-to-moderate acute pain, is paracetamol comparable to ibuprofen in terms of analgesic efficacy and safety?

PICO 3—Alternating therapy

In children with acute pain, does alternating paracetamol and ibuprofen improve pain control and reduce the need for treatment escalation compared to monotherapy?

PICO 4—Opioids vs. non-opioids

In children with severe acute pain, do opioid analgesics provide superior pain control compared to non-opioid treatments while maintaining an acceptable safety profile?

PICO 5—Intranasal opioids

In children requiring analgesia for acute pain in emergency settings, does intranasal opioid administration provide comparable efficacy and acceptability to intravenous administration?

PICO 6—Inhaled vs. topical analgesia

In children undergoing minor painful procedures, is inhaled analgesia faster and more effective than topical anesthetics?

PICO 7—Non-pharmacological interventions

In children undergoing painful procedures, do non-pharmacological interventions reduce pain, anxiety, and distress compared to standard care?

### 3.2. Literature Search

A total of 13 studies were included across seven PICO questions. Cohen’s k between reviewers was 0.86. The most robust evidence was available for PICO 1, 2, and 7. In particular, for PICO 3 and 6, no articles were identified, whereas for PICO 4 and 5, one article each was identified, on the basis of which it was possible to formulate clinical recommendations.

## 4. Summary of the Evidence

For PICO 1, 2, and 7, at least three eligible studies were identified; therefore, formal clinical recommendations were developed and assessed using the GRADE approach. For PICO 3–6, fewer than three eligible studies were available, or no studies were identified; therefore, the evidence was considered insufficient for formal grading, and only clinical guidance was provided when supported by available data.

### 4.1. PICO 1—Included Studies and Sample Characteristics

**Figure 1:**
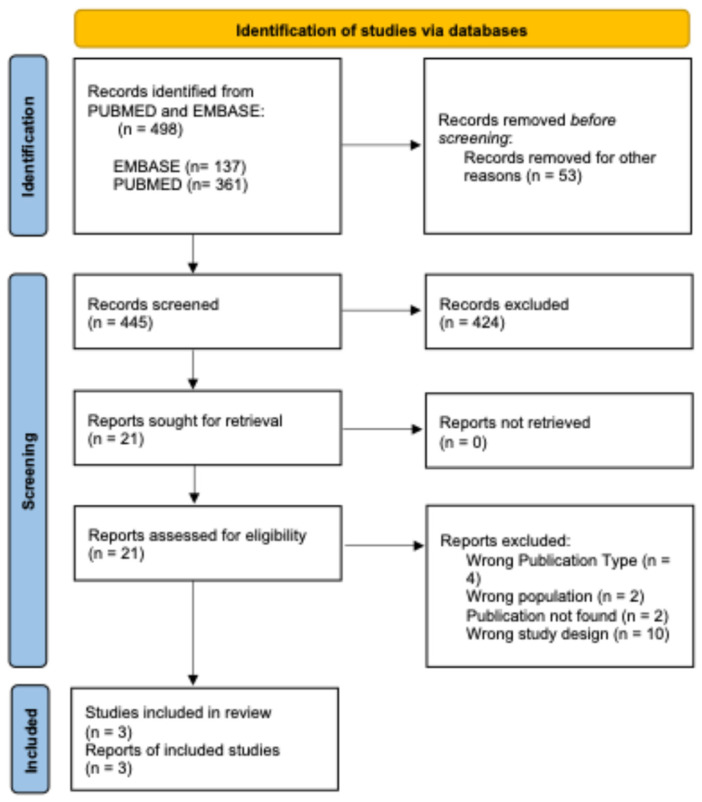
PRISMA 2020 flow diagram for PICO 1.

Three prospective observational studies were selected, evaluating reliability, validity, and response to analgesic administration using validated scales [14,15,16]. The literature flow chart for PICO 1 is given in Figure 1. The sample sizes ranged from >100 to over 700 children, aged up to 17 years, including emergency departments and hospital wards.

#### 4.1.1. PICO 1—Summary of Results

Validated scales showed high agreement in their use and a significant reduction in pain scores following analgesia (Table 1). None of the studies compared the use of scales with no scale use or directly measured prescribing appropriateness; therefore, such inferences cannot be quantified.

#### 4.1.2. PICO 1—Clinical Recommendations

Validated pain scales should be routinely used to support structured assessment of acute pain in children (moderate certainty). Evidence demonstrates good reliability and validity, although direct evidence that scale use improves prescribing appropriateness compared with unstructured assessment remains limited.

### 4.2. PICO 2—Included Studies and Sample Characteristics

**Figure 2:**
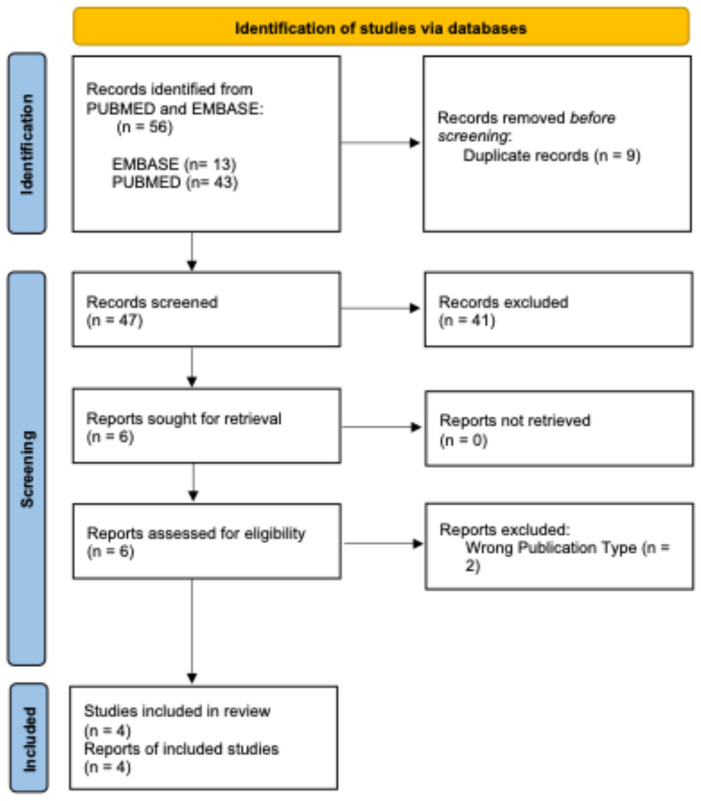
PRISMA 2020 flow diagram for PICO 2.

Four randomized trials conducted in dental settings (tooth extractions, pulpotomies, orthodontic procedures) were included (Figure 2), with sample sizes ranging from 45 to over 200 children aged 5–18 years [17,18,19,20].

#### 4.2.1. PICO 2—Summary of Results

Three studies reported superior efficacy of ibuprofen compared to paracetamol at specific time points (4–24 h post-procedure) [18,19,20], while one study showed equivalence between the two and no superiority over placebo [17]. The combination of paracetamol + ibuprofen was superior to single treatments in one large study. No evidence was available for otitis, pharyngitis, or minor trauma, as these were not included in the studies. Both drugs appear safe when used at recommended doses (Table 2).

#### 4.2.2. PICO 2—Clinical Recommendations

Both paracetamol and ibuprofen appear effective and safe at recommended doses for mild-to-moderate acute pediatric pain. Available evidence, mainly from dental settings, suggests comparable overall efficacy, although ibuprofen may provide better pain control at some later time points. Evidence is insufficient to recommend routine preference of one agent over the other across all clinical contexts (moderate certainty).

### 4.3. PICO 7—Included Studies and Sample Characteristics

**Figure 3:**
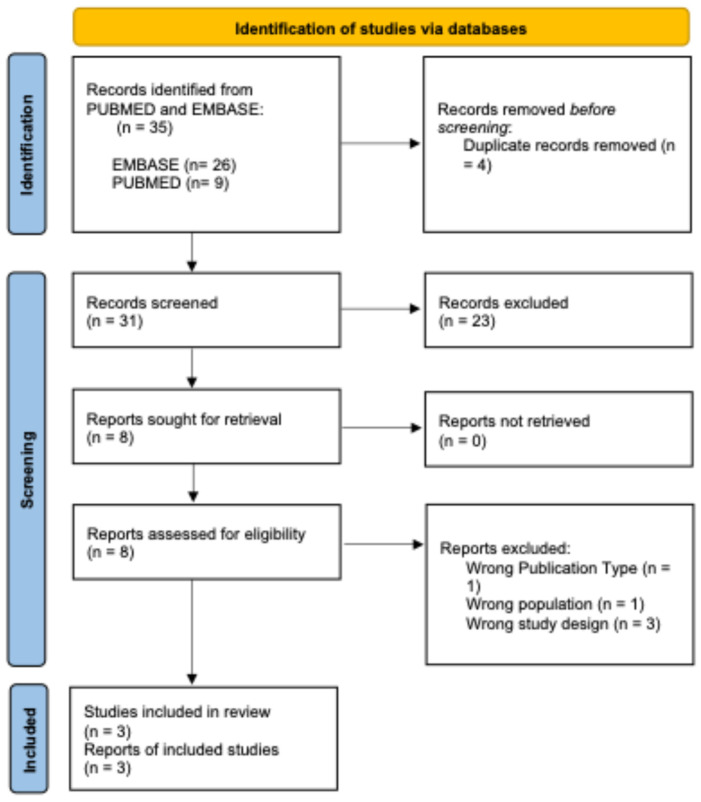
PRISMA 2020 flow diagram for PICO 7.

Three studies were included: two RCTs (robot distraction and virtual reality) [21,22] and one pre–post interventional study (hospital play) [23]. Sample sizes ranged from 53 to over 300 children undergoing procedures such as nasal endoscopy, peripheral venous access insertion, and hospital diagnostic-therapeutic procedures (Figure 3).

#### 4.3.1. Summary of Results

Virtual reality significantly reduced pain and anxiety compared to standard care. Robot distraction reduced observed distress and showed a trend toward pain reduction, although not statistically significant. Hospital play significantly reduced negative emotional behaviors during hospitalization. No adverse events were reported (Table 3).

#### 4.3.2. PICO 7—Clinical Recommendations

Non-pharmacological interventions should be considered adjunctive strategies rather than alternatives to pharmacological analgesia. Current evidence supports benefits on anxiety, distress, and procedural experience, while evidence for direct pain reduction remains limited and of low/very low certainty (low certainty).

#### 4.3.3. PICO 4—Summary of the Results

**Figure 4:**
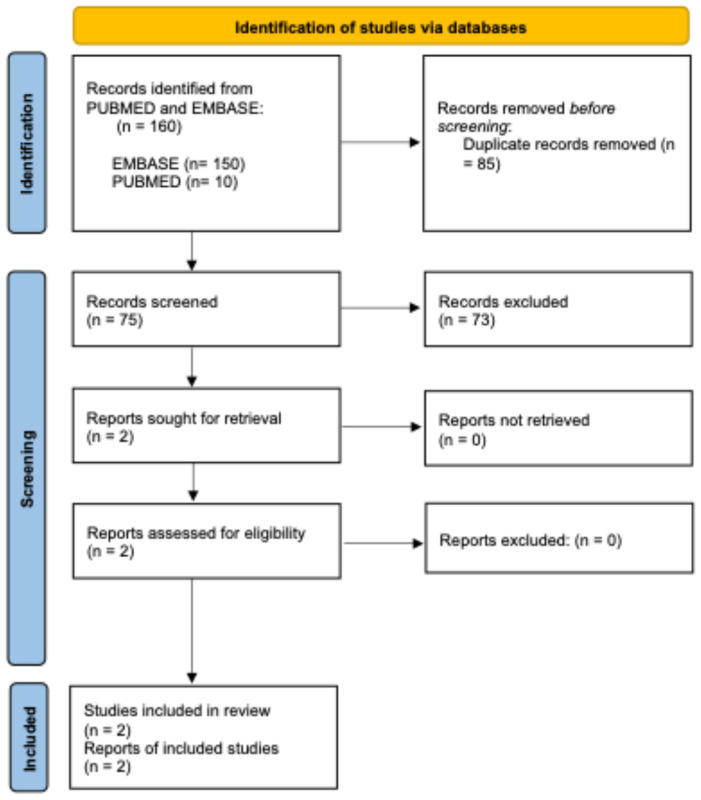
PRISMA 2020 flow diagram for PICO 4.

Two RCTs were identified as shown in Figure 4 [24,25]. The characteristics of these studies are summarized in Table 4.

#### 4.3.4. PICO 4—Clinical Guidance

Available evidence does not support routine opioid use as first-line treatment for clinically stable children with mild-to-moderate musculoskeletal or postoperative pain when non-opioid analgesics are appropriately used. Opioids should remain available for selected cases of moderate-to-severe pain not adequately controlled with first-line treatment.

#### 4.3.5. PICO 5—Summary of the Results

**Figure 5:**
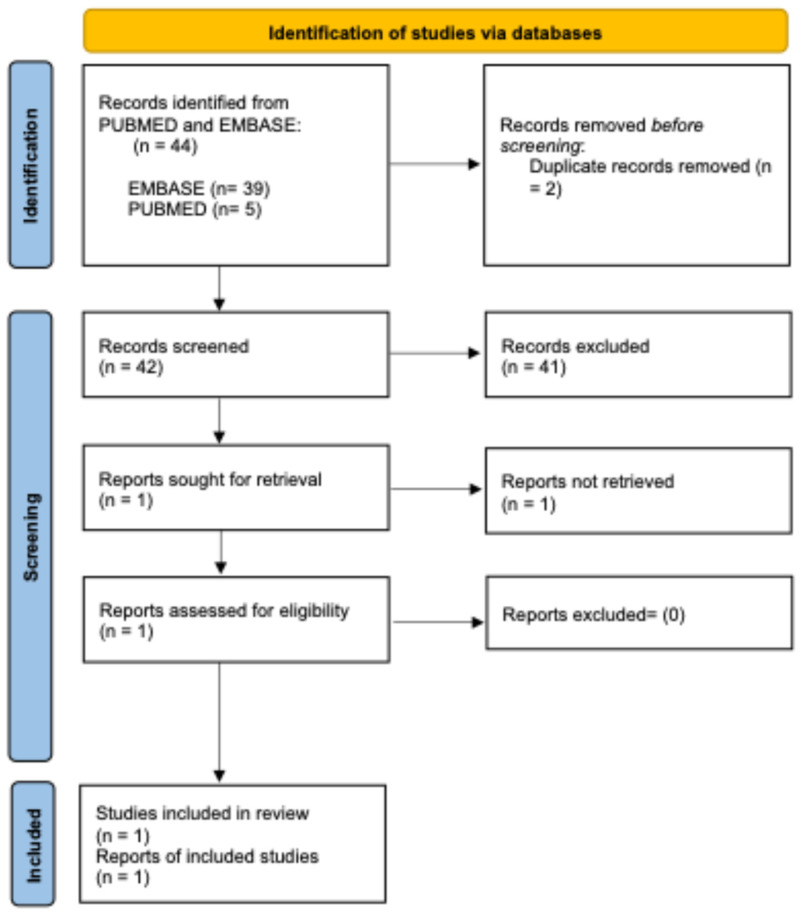
PRISMA 2020 flow diagram for PICO 5.

Only one RCT was identified, as shown in Figure 5 [26]. The characteristics of this study are summarized in Table 5.

#### 4.3.6. PICO 5—Clinical Guidance

In selected emergency procedural settings, intranasal fentanyl may be an effective and feasible alternative to intravenous morphine, particularly when venous access may delay analgesia. Evidence remains limited to short-term procedural outcomes.

### 4.4. PICO 3 and 6

**Figure 6:**
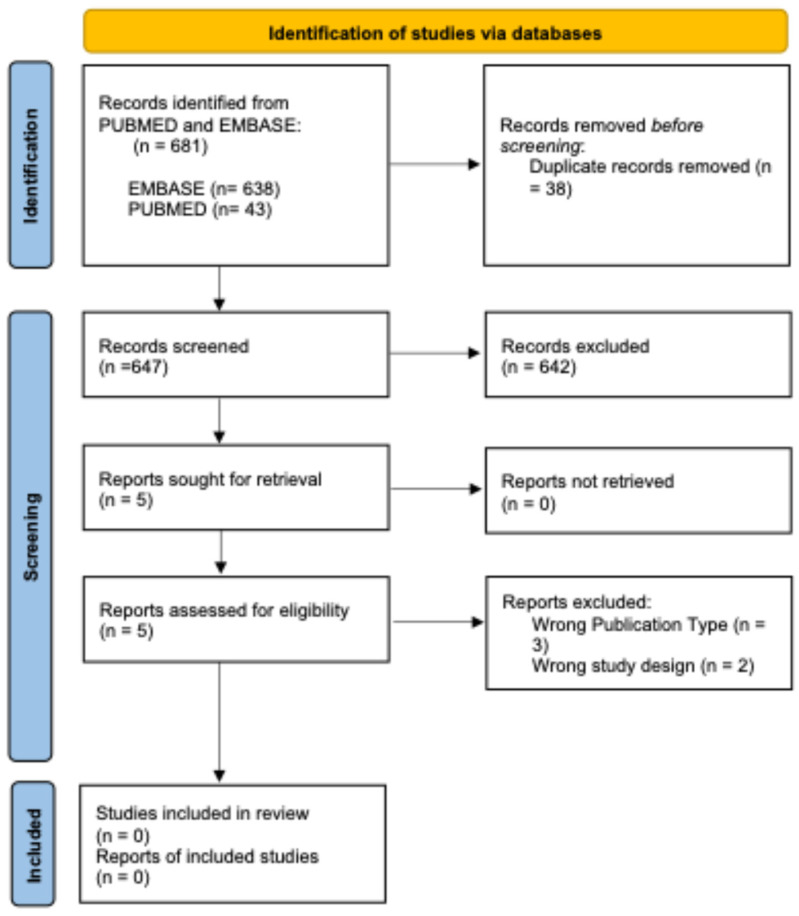
PRISMA 2020 flow diagram for PICO 3.

**Figure 7:**
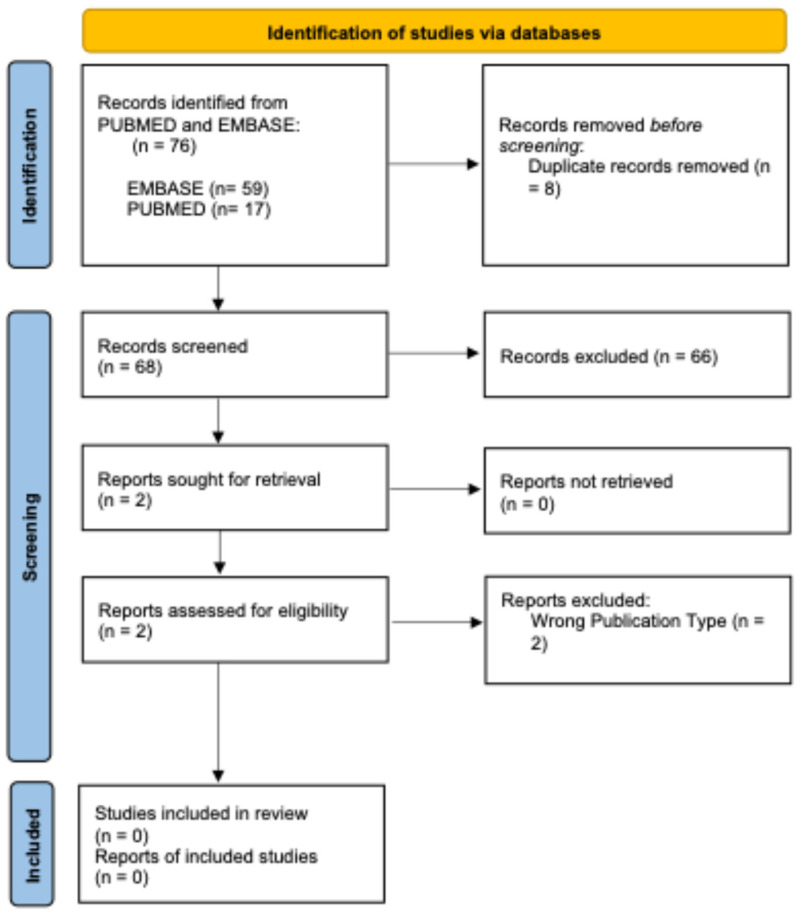
PRISMA 2020 flow diagram for PICO 6.

For PICO 3 and 6, no study was identified, as shown in Figure 6 and Figure 7, respectively.

### 4.5. Risk of Bias Assessment

Risk of bias assessment is reported in the Supplementary Material S2.

### 4.6. GRADE

GRADE analyses are detailed in the Supplementary Material S3.

## 5. Discussion

This work provides a structured synthesis of the available evidence on the management of acute pain in the pediatric population, addressing the topic through clinical questions formulated according to the PICO model and integrating data from observational and interventional studies. The results show that, despite the clinical relevance of acute pain in children and the availability of effective tools and therapeutic options, the scientific evidence remains heterogeneous and, in several areas, limited [1,2,27,28]. Moreover, most recommendations were supported by a relatively small number of studies, frequently conducted in highly specific clinical settings, particularly dental procedures and emergency departments. Consequently, the external validity and generalizability of some recommendations to other acute pain conditions should be interpreted with caution.

One of the main strengths identified concerns the importance of systematic pain assessment using validated scales (PICO 1). The included studies demonstrate high reliability and validity of assessment tools, as well as a clear association between the use of scales and reduction in pain scores following analgesic administration, confirming their importance in daily clinical practice [3]. Available consensus documents highlight that the routine use of standardized scales improves pain recognition and promotes more timely and appropriate analgesic intervention [29]. However, the lack of direct comparisons between structured scale use and the absence of formal assessment prevents quantification of the direct impact of these tools on prescribing appropriateness. Nevertheless, the data strongly support the systematic integration of pain assessment into routine clinical practice as an essential prerequisite for effective and timely management [1,2]. In the Italian context, some data have documented a progressive increase in the use of validated scales in hospital settings. However, significant gaps persist both in academic training and in systematic implementation in daily clinical practice, particularly in out-of-hospital and emergency settings [30,31]. This work further reinforces the need for structured and homogeneous implementation of pediatric acute pain assessment tools.

Regarding the pharmacological management of mild-to-moderate acute pain (PICO 2), the available evidence, although limited to specific clinical settings, suggests at least equivalent, and in some cases potentially superior, efficacy of ibuprofen compared with paracetamol. However, evidence derived from pediatric studies remains inconclusive and partly extrapolated from adult data [32]. A major gap is also represented by the evaluation of treatment adherence, particularly in the postoperative setting, both in hospitals and among parents and patients. The absence of includable studies from other common clinical contexts (such as pharyngitis or minor trauma) represents a significant limitation of the literature, restricting the generalizability of findings. Accordingly, evidence derived predominantly from dental pain should not be automatically extrapolated to other causes of acute pediatric pain, and recommendations should always be interpreted within the specific clinical context. This highlights the need for pragmatic studies conducted in clinical settings more representative of everyday pediatric practice. Combination therapy with paracetamol and ibuprofen, already used in some clinical contexts, shows promising results in terms of improved pain control and reduced need for rescue medication [33]. However, RCTs conducted outside the dental setting (e.g., emergency department pain or post-tonsillectomy pain) have shown conflicting results, and some did not demonstrate superiority of combination therapy over single agents [34,35,36]. In the absence of robust pediatric evidence specifically designed to assess short- and medium-term safety and efficacy, particularly regarding potential renal harm [33], several authors recommend caution and a use guided by clinical judgment [37].

Another area of interest concerns non-pharmacological interventions in procedural pain (PICO 7). Available evidence indicates that strategies such as virtual reality, robot-assisted distraction, and hospital play can reduce pain, anxiety, and behavioral distress in children undergoing painful procedures, without reported adverse events. Although the number of studies is limited and outcomes are heterogeneous, these interventions appear promising and consistent with a multimodal approach to pediatric pain management, in line with the principles of humanization of care [38,39].

For PICO 3–6, the analysis revealed a significant lack of evidence. In particular, no eligible studies were identified for PICO 3 and 6, while only one study was included for each of PICO 4 and 5. In this context, it was not possible to formulate formal recommendations or perform a GRADE assessment; however, based on the available evidence, clinical guidance useful for practice was developed. Specifically, for PICO 4, the data suggest that in pediatric acute pain, particularly musculoskeletal or postoperative pain, non-opioid analgesics are often effective, with no clear additional benefit from the routine use of opioids. This finding is particularly relevant in light of growing concerns regarding opioid use in pediatric populations and supports a cautious and selective approach, reserving opioids for cases of moderate-to-severe pain not adequately controlled with first-line therapies [40,41].

Regarding PICO 5, available evidence suggests that intranasal fentanyl may represent a valid alternative to intravenous morphine in procedural pain in the emergency department, offering advantages in terms of rapid administration, patient comfort, and clinical feasibility, consistent with previous literature [42,43]. However, these findings derive from highly selected settings with short-term follow-up, and their use should therefore be guided by clinical judgment and well-defined local protocols.

From a broader public health perspective, pain-related conditions remain among the leading contributors to disability worldwide, highlighting the importance of developing rigorous, evidence-informed approaches for pain assessment and management across healthcare settings [44,45]. The observed limitations in the evidence base are likely not incidental but reflect structural challenges inherent to pediatric pain research. Ethical constraints, heterogeneity of clinical presentations, and the difficulty of applying standardized and age-appropriate outcome measures limit the feasibility of large, well-controlled randomized trials. In addition, acute pain in children encompasses diverse conditions (procedural, traumatic, infectious), further complicating the generation of homogeneous and comparable datasets [2,46]. These factors contribute to the fragmented and uneven nature of the available evidence. It is also worth noting that the present findings should be interpreted in light of the methodology used to formulate the recommendations. Formal GRADE recommendations were developed only for PICO questions in which the available evidence was considered sufficient to permit a structured assessment of certainty. Importantly, the GRADE approach considers not only the number of available studies but also their methodological quality, consistency, directness, precision, and risk of publication bias. Consequently, moderate-certainty recommendations may be appropriate despite a relatively limited evidence base. Conversely, for PICOs with insufficient evidence, only evidence-informed clinical guidance statements were developed.

## 6. Limitations

This study has several limitations that should be considered when interpreting the findings. First, the overall body of evidence is limited in both quantity and quality for several of the PICO questions. While sufficient data were available to support formal GRADE-based recommendations for some domains (PICOs 1, 2, and 7), other areas (PICOs 3–6) were characterized by a paucity of eligible studies, with some PICOs lacking any primary evidence. This restricted the ability to perform robust evidence synthesis and prevented formal grading, leading instead to the formulation of guidance based on limited data and expert interpretation. However, this analysis points out several areas needing additional research. Second, there was substantial heterogeneity across included studies in terms of study design, patient populations, interventions, and outcome measures. This variability limited the comparability of results and, in many cases, precluded quantitative synthesis through meta-analysis. As a result, conclusions are largely based on qualitative synthesis, which may be more prone to interpretative bias. Third, the evidence base was often derived from highly specific clinical contexts, such as dental procedures or emergency department settings. The lack of studies conducted in more common and diverse pediatric scenarios (e.g., minor trauma, infectious conditions, or primary care settings) reduces the generalizability of the findings to routine clinical practice. Fourth, the included studies frequently had relatively small sample sizes and were not always powered to detect clinically relevant differences or rare adverse events, particularly in the evaluation of pharmacological interventions such as opioids. This limitation affects the precision of estimates and the ability to draw firm conclusions regarding safety profiles. Fifth, important outcomes such as long-term effectiveness, functional recovery, patient-reported outcomes, and treatment adherence were inconsistently reported or not assessed. In particular, adherence to analgesic therapy, especially in outpatient and postoperative settings, remains an underexplored area, despite its potential impact on treatment effectiveness. Sixth, the methodological quality of the included studies varied, with potential risks of bias related to study design, outcome assessment, and reporting. Although standardized tools were used to assess risk of bias, residual confounding and bias cannot be excluded. Seventh, this review excluded non-English publications and gray literature, which may have introduced language publication bias and resulted in the omission of potentially relevant earlier studies. Finally, an important limitation concerns the lack of evidence specifically addressing children with chronic underlying conditions or comorbidities. Most included studies focused on otherwise healthy pediatric populations, and therefore the applicability of the findings to patients with chronic diseases (e.g., neurological, oncological, or complex medical conditions) is uncertain. In these populations, pain mechanisms, treatment responses, and risk–benefit profiles may differ substantially, limiting the generalizability of the available evidence [47]. Finally, the review protocol was not prospectively registered (e.g., PROSPERO). Although this work incorporated a systematic review of the literature, its primary objective was the development of evidence-based recommendations and clinical guidance through a structured expert consensus process rather than the conduct of a standalone systematic review. Accordingly, a predefined methodological protocol was established before study initiation and was applied consistently throughout the review and recommendation development process.

## 7. Conclusions

Current evidence supports the routine use of structured pain assessment and multimodal approaches for pediatric acute pain management. Validated pain assessment tools should be routinely implemented in clinical practice, and both pharmacological and non-pharmacological strategies should be integrated according to the child’s clinical condition and procedural context. Paracetamol and ibuprofen remain appropriate first-line options for mild-to-moderate acute pain, although some evidence suggests context-specific advantages of ibuprofen or combination therapy. However, several recommendations are supported by relatively limited evidence derived from small studies conducted in specific clinical settings. Accordingly, the present recommendations should be interpreted as evidence-informed guidance reflecting the current state of the literature rather than definitive standards of care, and should be applied according to the individual clinical context. As new pediatric evidence becomes available, these recommendations should be updated. Further well-designed pediatric studies across a broader range of acute pain conditions and healthcare settings are needed to define optimal treatment strategies, clarify safety profiles, and support stronger evidence-based recommendations.

Acute pediatric pain should be managed through structured, age-appropriate assessment and a multimodal strategy that combines timely pharmacological treatment with non-pharmacological support. Validated pain scales should be used routinely, while paracetamol and ibuprofen remain appropriate first-line options for mild-to-moderate pain. Non-pharmacological interventions, including virtual reality, distraction, and hospital play, may reduce anxiety and procedural distress and are best considered as adjuncts to, rather than substitutes for, effective analgesia. However, the certainty of evidence for these approaches is low, and the available data are heterogeneous. Evidence regarding opioids is also limited and does not demonstrate consistent superiority over non-opioid strategies; opioids should therefore be reserved for carefully selected children with moderate-to-severe pain that remains inadequately controlled after first-line treatment. Intranasal fentanyl appears clinically feasible in emergency settings, but further comparative studies are needed to define its effectiveness, safety, acceptability, and optimal place in care. Important evidence gaps also remain for alternating regimens and for direct comparisons between inhaled and topical analgesia in specific procedural settings.

Future research should prioritize adequately powered, multicenter studies with standardized pain, distress, functional, safety, and rescue-medication outcomes. Because pediatric acute pain research presents substantial ethical challenges, study designs should ensure that all participants receive appropriate baseline analgesia. When an effective standard treatment exists, untreated or pure-placebo control groups should generally be avoided in favor of active-control designs using established therapies such as acetaminophen or ibuprofen. Protocols should include predefined rescue analgesia and explicit criteria for immediate treatment escalation when pain remains uncontrolled. Add-on designs are particularly suitable for evaluating non-pharmacological interventions: all children receive standard analgesic care, and the investigational intervention is added in one group and compared with standard care alone. This approach preserves clinical equipoise, reduces ethical concerns, and permits evaluation of outcomes that are meaningful to patients and clinicians, such as faster pain relief, lower rescue-medication requirements, reduced distress, improved function, and fewer adverse effects. The central research objective should be to determine how to relieve pain rapidly, safely, and intelligently through optimized multimodal combinations—not to expose children to avoidable pain for methodological purposes. Aligning scientific rigor with beneficence will strengthen the evidence base, facilitate ethical approval, and increase the clinical relevance of future pediatric acute pain studies.

## Figures and Tables

**Table 1:** Study Characteristics of PICO 1.

Authors, Year, Country	Study Design and Setting	Duration	Inclusion/Exclusion Criteria	Underlying Condition	Sample Size	Scale Used	Main Results
Abu-Saad H.H. et al., 1994 [14], The Netherlands	Prospective observational study, 2 centers involved, pediatric wards	Study 1: 4 h; Study 2: single measurement	Inclusion: Children aged 5–15 years hospitalized for medical or surgical procedures, without intellectual disability and with prescribed analgesic drugs. Exclusion: none	Study 1: Postoperative pain (general, orthopedic, plastic, thoracic surgery, trauma, urology, cancer). Study 2: Post-procedural pain (medical or surgical)	105 (26 in Study 1; 79 in Study 2)	Abu-Saad Paediatric Pain Assessment Tool	Post-analgesia pain scores were significantly lower than pre-analgesia scores.
Bailey B. et al., 2010 [15], Canada	Cross-sectional study, single center, emergency department	40–50 min (assessments at T0, T20, and T40–50 min post-analgesia)	Inclusion: Children aged 4–17 years; emergency department presentation with acute pain.	Various	733 (760 enrolled, 27 excluded)	Verbal Numeric Scale (vNRS). Comparator scales: Faces Pain Scale–Revised (FPS-R)	Convergent validity, known-groups validity, responsivity and reliability of the vNRS were strong in children 6–17 years of age. Convergent validity was not strong in 4- and 5-year-olds.
Bringuier S. et al., 2009 [16], France	Prospective observational, single-center, pediatric surgery center	Single assessment period (but 4 distinct peri-operative timepoints measured)	Inclusion: Children aged 1–7 years; surgery under general anesthesia; stay at hospital for at least 24 h. Exclusion: emergency, outpatients, chronic pain, intellectual disability.	Orthopedic (43.2%), Plastic (30.7%), Abdominal (16.2%), Urologic (10.1%)	150 (2 dropouts)	Behavioral Rating Scales: CHEOPS, CHIPPS, FLACC, OPS	Internal validities were excellent but external validities mixed. FLACC had the highest sensitivity and specificity. Silence was a high-risk factor for false negative evaluations.

**Table 2:** Study Characteristics of PICO 2.

Authors, Year, Country	Study Design and Setting	Duration	Inclusion/Exclusion Criteria	Underlying Condition	Sample Size	Intervention	Comparator	Outcomes	Main Results
Salmassian R. et al., 2009 [17], USA	Randomized, double-blind, single-center controlled trial; orthodontic clinic	7 days	Orthodontic treatment planned; healthy; no recent antibiotics/analgesics; age 12–18 years	Need for fixed orthodontic treatment	60 (21 acetaminophen, 19 ibuprofen, 20 placebo)	Acetaminophen 600 mg or ibuprofen 400 mg orally at 9 time points over 7 days	Placebo	Pain intensity	No statistically significant differences in pain control among acetaminophen, ibuprofen, and placebo.
Baygin O. et al., 2011 [18], Türkiye	Randomized, double-blind, single-center controlled trial; dental outpatient clinic	24 h	Children 6–12 years; ASA I; primary mandibular molar extraction; no drug allergy	Primary mandibular molar extraction	45 (15 per group)	Single preoperative dose: Paracetamol 250 mg/5 mL or ibuprofen 100 mg/5 mL	Placebo	Postoperative pain scores	Both drugs showed significantly lower pain scores compared to placebo. Ibuprofen was superior to paracetamol at 15 min and 4 h.
Abou El Fadl R. et al., 2019 [19], Egypt	Randomized, double-blind, single-center controlled trial; dental department	During treatment	Children 7–9 years; cooperative; carious mandibular second primary molar indicated for pulpotomy	Caries in primary mandibular molars	60 (20 per group)	Single preoperative dose: Acetaminophen 200 mg/5 mL or ibuprofen 100 mg/5 mL	Placebo	Pain scores during access cavity preparation	Both analgesics showed no clinical advantage over placebo in increasing the efficacy of buccal infiltration.
Gazal G. et al., 2007 [20], UK	Randomized, double-blind, single-center controlled trial; dental hospital	15 min post-op	Children 2–12 years; ASA I–II; elective extraction of 1–14 teeth under general anesthesia	Dental extractions under GA	201 (47 ibuprofen, 51 paracetamol/ibuprofen, 55 usual-dose paracetamol, 48 high-dose paracetamol)	Preoperative dose: Ibuprofen alone (5 mg/kg), paracetamol/ibuprofen combination (15/5 mg/kg), high-dose paracetamol (20 mg/kg)	Usual-dose paracetamol (15 mg/kg) as control	Pain and distress scores	Significant decreases in mean pain and distress scores for both the ibuprofen alone and paracetamol/ibuprofen combination groups compared to the usual-dose paracetamol group at 15 min postoperatively.

**Table 3:** Study Characteristics of PICO 7.

Authors, Year, Country	Study Design and Setting	Duration	Inclusion/Exclusion Criteria	Underlying Condition	Sample Size	Intervention vs. Comparator	Outcomes	Main Results
Li W.H.C. et al., 2016 [23], Hong Kong	Quasi-experimental study (non-equivalent control group pre/post-test); two public hospitals	30 min/day	Inclusion: Chinese children 3–12 years; hospitalization for ≥3 consecutive days. Exclusion: Cognitive/learning difficulties.	Hospitalization for medical treatments/investigations	304 (154 intervention, 150 usual care)	Intervention: Hospital play interventions (30 min/day). Comparator: Usual care.	Anxiety and negative emotions (CEMS)	Children receiving the intervention exhibited significantly fewer negative emotions (*p* < 0.001) and lower anxiety levels (*p* < 0.005) compared with usual care.
Liu K.Y. et al., 2020 [21], USA	Randomized controlled trial, single-center; outpatient otolaryngology clinic	During nasal endoscopy	Inclusion: 7–17 years undergoing nasal endoscopy. Exclusion: Chronic pain, developmental delay, neurological/seizure disorders, visual impairment.	Nasal endoscopy	53 (30 VR, 23 control)	Intervention: VR game via Oculus Go + standard topical analgesia. Comparator: Standard of care + topical analgesia.	Pain (FACES), Anxiety (SUDS), Distress (CEMS), Satisfaction	VR significantly reduced pain (*p* = 0.018), anxiety (*p* = 0.0002), and distress (*p* = 0.0001), and increased satisfaction compared to control. No adverse effects reported.
Ali S. et al., 2021 [22], Canada	Randomized controlled trial, single-center; pediatric emergency department	During IV insertion	Inclusion: 6–11 years requiring IV cannulation. Exclusion: Sensory/visual/hearing impairments, neurocognitive delay, altered pain sensitivity.	Intravenous cannulation	86 (43 robot, 43 standard care)	Intervention: Humanoid robot-based distraction + standard care. Comparator: Standard care.	Distress (OSBD-R), Pain (FPS-R), Parental anxiety (STAI-S)	Robot significantly reduced observed child distress (*p* < 0.05) and parental anxiety (*p* = 0.04). There was no statistically significant difference in child-reported pain (*p* = 0.13).

**Table 4:** Study Characteristics of PICO 4.

Authors, Year, Country	Study Design and Setting	Duration	Inclusion/Exclusion Criteria	Underlying Condition	Sample Size	Comparison	Primary Outcomes/Results
Le May et al., 2017 [24], Canada	Randomized, double-blind controlled clinical trial; 3 EDs	120 min	Inclusion: Patients aged 6–17 years presenting to the ED with a non-deformed, non–neurovascularly compromised upper or lower limb musculoskeletal injury; self-reported VAS pain > 29 mm; ability to communicate in English or French. Exclusion: Allergy to morphine or ibuprofen; suspected child abuse; inability to self-report pain; chronic pain requiring daily analgesics; NSAID or opioid use within 3 h; injury to >1 limb; hepatic or renal disease; bleeding disorders; neurocognitive disability; history of sleep apnea or loud snoring within 5 days.	Limb musculoskeletal injury	Morphine + ibuprofen (n = 201); Morphine + placebo (n = 201); Ibuprofen + placebo (n = 99).	Morphine vs. ibuprofen vs. combination therapy	No significant difference between groups in achieving VAS < 30 mm at T−60 (*p* = 0.81). All groups showed pain reduction over time; statistically significant only at T−120 (*p* = 0.02).
Lu et al., 2025 [25], USA	Randomized controlled clinical trial: tertiary children’s hospital	14 days	Exclusion: Down syndrome; coagulopathy; craniofacial anomalies (except plagiocephaly or submucosal cleft palate); chronic opioid use; pregnancy; contraindications to study drugs; inability to communicate/localize pain; foster care placement. Caregivers required English proficiency.	Adenotonsillectomy	36 enrolled (25 analyzed); 86% aged 3–7 years, 14% aged 8–12 years	Oxycodone vs. acetaminophen and/or ibuprofen	Mean postoperative pain score over 14 days measured using the Wong–Baker FACES scale.

**Table 5:** Study Characteristics of PICO 5.

Authors, Year, Country	Study Design and Setting	Duration	Inclusion/Exclusion Criteria	Underlying Condition	Sample Size	Comparison	Primary Outcomes/Results
Fenster et al., 2018 [26], USA	Randomized trial comparing intranasal fentanyl with intravenous morphine for abscess incision and drainage	10 min post-procedure	Inclusion: 4–18 years; cutaneous abscess requiring incision and drainage. Exclusion: Developmental delay, chronic pain, allergy to study drug, opioid receipt within 4 h, moderate/deep sedation required.	Cutaneous abscess	20 participants analyzed (10 per group)	Intranasal fentanyl (2 μg/kg) vs. Intravenous morphine (0.1 mg/kg)	IN fentanyl was noninferior (and potentially superior) to IV morphine for reducing pain and distress (difference in OSBD-R: −13.45). In total, 4 patients (40%) receiving IV morphine had treatment failures (excessive pain/required sedation) vs. 0% in IN fentanyl.

## Data Availability

No new data were created or analyzed in this study. Data sharing is not applicable to this article.

## Supplementary Materials

The following supporting information can be downloaded at https://www.mdpi.com/article/10.3390/jcm15155802/s1. Supplementary Material S1: Full search strategies for each PICO question; Supplementary Material S2: Risk of bias assessment; Supplementary Material S3: GRADE Analyses for each PICO question; Supplementary Material S4: PRISMA checklist 2020.
