# Supplementary material for: Acute Pain Management in Children: A Systematic Review and GRADE-Based Recommendations
Source: J Clin Med. 2026 Jul 24;15(15):5802. doi: 10.3390/jcm15155802 (PMC13467242; doi:10.3390/jcm15155802)
Supplement: Supplementary file 1 [file jcm-15-05802-s001.zip › jcm-4442532-supplementary.pdf]

Supplementary Material S1. Full search strategies for each PICO question

Clinical Question	Research Question	PICO Summary
**1**	In children with acute pain, does the use of validated pain assessment scales improve pain identification and management compared with unstructured clinical observation alone?	P: Children and adolescents (1 month–18 years) with acute pain (trauma, infections, post-procedural)<br>I: Validated age-appropriate scales (FLACC, Wong–Baker, NRS)<br>C: Unstructured clinical observation<br>O: Accuracy, appropriateness and timeliness of analgesia, satisfaction
**2**	In children and adolescents with mild–moderate acute pain, does paracetamol have comparable efficacy and safety to ibuprofen?	P: Children and adolescents (1 month–18 years) with mild–moderate acute pain<br>I: Paracetamol<br>C: Ibuprofen<br>O: Analgesic efficacy, adverse events
**3**	In children with mild–moderate acute pain, does alternating paracetamol and ibuprofen improve pain control versus monotherapy?	P: Children and adolescents with mild–moderate acute pain<br>I: Alternating paracetamol/ibuprofen<br>C: Monotherapy<br>O: Pain control, escalation, safety, adherence
**4**	In children with severe acute pain, do opioids provide better analgesia than NSAIDs/paracetamol with acceptable safety?	P: Children and adolescents with severe acute pain<br>I: Systemic opioids<br>C: NSAIDs/paracetamol<br>O: Pain reduction, adverse events
**5**	In children with severe acute pain in emergency settings, are intranasal	P: Children in emergency care<br>I: Intranasal opioids<br>C: IV opioids

	opioids comparable to IV opioids?	O: Onset, efficacy, acceptability, safety
**6**	In children undergoing minor painful procedures, is inhaled analgesia faster and more effective than topical anesthetics?	P: Children undergoing minor procedures<br>I: Inhaled analgesia<br>C: Topical anesthetics<br>O: Onset, efficacy, acceptability
**7**	Are non-pharmacological techniques as effective as pharmacological analgesia during minor procedures in children?	P: Children undergoing painful minor procedures<br>I: Non-pharmacological techniques<br>C: Pharmacological analgesia<br>O: Pain, anxiety, adverse effects

**PICO**	**PUBMED**	**EMBASE**
1	(("Child"[Mesh] OR "Adolescent"[Mesh] OR "Pediatrics"[Mesh] OR child* OR pediatric* OR adolescent* OR toddler*)<br>AND<br>("Pain, Acute"[Mesh] OR "Pain"[Mesh] OR "Acute Pain" OR "procedural pain" OR "trauma" OR "infection" OR "postoperative pain" OR<br>"Pain Measurement"[Mesh] OR "Pain scale" OR "pain assessment" OR "validated scale" OR "FLACC" OR "Wong-Baker" OR "NRS")<br>AND<br>("Clinical Observation" OR "unstructured clinical observation" OR "clinical judgment" OR "non-standardized assessment" OR "Diagnostic Accuracy" OR "Sensitivity and Specificity"[Mesh] OR "Observer Variation"[Mesh] OR "pain detection" OR "pain recognition")<br>AND	('child'/exp OR 'adolescent'/exp OR pediatric*:ti,ab OR child*:ti,ab OR toddler*:ti,ab OR adolescent*:ti,ab)<br>AND<br>('acute pain'/exp OR 'procedural pain':ti,ab OR 'trauma':ti,ab OR 'infection':ti,ab OR 'postoperative pain':ti,ab)<br>AND<br>('pain assessment'/exp OR 'pain rating scale'/exp OR 'validated scale':ti,ab OR 'pain scale':ti,ab OR 'flacc':ti,ab OR 'wong-baker':ti,ab OR 'nrs':ti,ab)<br>AND<br>('clinical observation':ti,ab OR 'unstructured observation':ti,ab OR 'clinical judgment':ti,ab OR 'non-standardized assessment':ti,ab)<br>AND<br>('diagnostic accuracy'/exp OR 'observer variation'/exp OR 'pain recognition':ti,ab OR 'pain detection':ti,ab)

	("Analgesics"[Mesh] OR "Pain Management"[Mesh] OR "timely intervention" OR "adequate analgesia" OR "analgesic treatment" OR "Patient Satisfaction"[Mesh] OR "Caregivers"[Mesh] OR "satisfaction" OR "caregiver satisfaction”)	AND<br>('pain management'/exp OR 'analgesic agent'/exp OR 'timely intervention':ti,ab OR 'adequate analgesia':ti,ab)<br>AND<br>('patient satisfaction'/exp OR 'caregiver'/exp OR satisfaction:ti,ab OR 'caregiver satisfaction':ti,ab)
2	(("Child"[Mesh] OR "Adolescent"[Mesh] OR "Pediatrics"[Mesh] OR child* OR pediatric* OR adolescent*)<br>AND<br>("Pain, Acute"[Mesh] OR "Pain"[Mesh] OR "Mild to Moderate Pain" OR "acute pain" OR "otitis media"[Mesh] OR "Pharyngitis"[Mesh] OR "musculoskeletal trauma")<br>AND<br>("Acetaminophen"[Mesh] OR paracetamol OR acetaminophen)<br>AND<br>("Ibuprofen"[Mesh] OR ibuprofen)<br>AND<br>("Pain Measurement"[Mesh] OR "Pain scale" OR "pain assessment" OR "FLACC" OR "Wong-Baker")<br>AND<br>("Adverse Effects"[Mesh] OR "Drug-Related Side Effects and Adverse Reactions"[Mesh] OR "Treatment Outcome"[Mesh] OR "Safety"[Mesh] OR "side effect" OR "adverse event" OR "complication"))	('child'/exp OR 'adolescent'/exp OR pediatric*:ti,ab OR child*:ti,ab OR adolescent*:ti,ab)<br>AND<br>('acute pain'/exp OR 'mild to moderate pain':ti,ab OR 'otitis media'/exp OR 'pharyngitis'/exp OR 'minor trauma':ti,ab OR 'musculoskeletal trauma':ti,ab)<br>AND<br>('paracetamol'/exp OR paracetamol:ti,ab OR acetaminophen:ti,ab)<br>AND<br>('ibuprofen'/exp OR ibuprofen:ti,ab)<br>AND<br>('pain assessment'/exp OR 'pain rating scale'/exp OR 'pain scale':ti,ab OR 'flacc':ti,ab OR 'wong-baker':ti,ab OR 'nrs':ti,ab)<br>AND<br>('adverse drug reaction'/exp OR 'side effect'/exp OR 'safety'/exp OR 'adverse event':ti,ab OR 'treatment outcome':ti,ab)
3<br>•	(("Child"[Mesh] OR "Adolescent"[Mesh] OR "Pediatrics"[Mesh] OR child* OR pediatric* OR adolescent*)<br>AND<br>("Pain, Acute"[Mesh] OR "Pain"[Mesh] OR "Mild to Moderate Pain" OR "acute pain")<br>AND<br>("Drug Administration Schedule"[Mesh] OR "alternating therapy" OR "alternating ibuprofen and acetaminophen" OR "combined regimen" OR "Pain	('child'/exp OR 'adolescent'/exp OR child*:ti,ab OR pediatric*:ti,ab OR adolescent*:ti,ab)<br>AND<br>('acute pain'/exp OR 'mild to moderate pain':ti,ab OR 'acute pain':ti,ab)<br>AND<br>(('paracetamol'/exp OR paracetamol:ti,ab OR acetaminophen:ti,ab) AND ('ibuprofen'/exp OR ibuprofen:ti,ab))<br>AND

	Measurement"[Mesh] OR "pain control" OR "pain relief" OR "pain assessment")<br>AND<br>(("Acetaminophen"[Mesh] OR paracetamol OR acetaminophen)<br>AND ("Ibuprofen"[Mesh] OR ibuprofen OR "Analgesics, Opioid"[Mesh] OR "Therapeutic Escalation" OR "hospital admission" OR "rescue medication")<br>AND<br>("Adverse Effects"[Mesh] OR "Drug-Related Side Effects and Adverse Reactions"[Mesh] OR "Safety"[Mesh] OR "side effect" OR "adverse event")<br>AND<br>("Medication Adherence"[Mesh] OR "Patient Compliance"[Mesh] OR "tolerability" OR "adherence" OR "treatment acceptance"))	('drug administration schedule'/exp OR 'alternating therapy':ti,ab OR 'combined analgesic regimen':ti,ab OR 'alternating ibuprofen and paracetamol':ti,ab)<br>AND<br>('pain assessment'/exp OR 'pain control':ti,ab OR 'pain relief':ti,ab)<br>AND<br>('opioid analgesic agent'/exp OR 'treatment escalation':ti,ab OR 'hospital admission':ti,ab OR 'rescue medication':ti,ab)<br>AND<br>('adverse drug reaction'/exp OR 'safety'/exp OR 'side effect':ti,ab OR 'adverse event':ti,ab)<br>AND<br>('treatment compliance'/exp OR 'treatment adherence':ti,ab OR 'tolerability':ti,ab OR 'patient compliance':ti,ab))
4	(("Child"[Mesh] OR "Adolescent"[Mesh] OR "Pediatrics"[Mesh] OR child* OR pediatric* OR adolescent*)<br>AND<br>("Pain, Acute"[Mesh] OR "Severe Pain" OR "acute severe pain" OR "fracture"[Mesh] OR "burns"[Mesh])<br>AND<br>("Analgesics, Opioid"[Mesh] OR "Morphine"[Mesh] OR "Oxycodone"[Mesh] OR opioid* OR morphine OR oxycodone)<br>AND<br>(("Anti-Inflammatory Agents, Non-Steroidal"[Mesh] OR "NSAIDs" OR "Non-Steroidal Anti-Inflammatory Drugs" OR ibuprofen OR naproxen)<br>OR<br>("Acetaminophen"[Mesh] OR paracetamol OR acetaminophen))<br>AND<br>("Pain Measurement"[Mesh] OR "Pain scale" OR "validated pain scale")<br>AND<br>("Adverse Effects"[Mesh] OR "Drug-Related Side Effects and	('child'/exp OR 'adolescent'/exp OR pediatric*:ti,ab OR child*:ti,ab OR adolescent*:ti,ab)<br>AND<br>('acute pain'/exp OR 'severe pain':ti,ab OR 'fracture'/exp OR 'burn'/exp)<br>AND<br>('opioid analgesic agent'/exp OR 'morphine'/exp OR 'oxycodone'/exp OR opioid*:ti,ab OR morphine:ti,ab OR oxycodone:ti,ab)<br>AND<br>(('non steroidal anti inflammatory drug'/exp OR 'NSAID'/exp OR 'ibuprofen'/exp OR 'naproxen'/exp OR 'naproxen':ti,ab OR 'ibuprofen':ti,ab)<br>OR<br>('paracetamol'/exp OR paracetamol:ti,ab OR acetaminophen:ti,ab))<br>AND<br>('pain measurement'/exp OR 'pain scale'/exp OR 'validated pain scale':ti,ab)<br>AND

	Adverse Reactions"[Mesh] OR "Safety"[Mesh] OR "side effect" OR "adverse event"))	('adverse drug reaction'/exp OR 'side effect'/exp OR 'safety'/exp OR 'adverse event':ti,ab)
5	(("Child"[Mesh] OR "Adolescent"[Mesh] OR "Pediatrics"[Mesh] OR child* OR pediatric* OR adolescent*) AND ("Pain, Acute"[Mesh] OR "Severe Pain" OR "acute severe pain" OR "pain") AND ("Analgesics, Opioid"[Mesh] OR opioid* OR fentanyl OR morphin*) AND (("Administration, Intranasal"[Mesh] OR "intranasal administration" OR "intranasal" OR "nasal spray" OR "intranasal route" OR "nasal" OR "Administration, Intravenous"[Mesh] OR "intravenous administration" OR "intravenous" OR "IV" AND ("efficacy" OR "effectiveness" "acceptance" OR "acceptability" OR "comfort" OR "ease of use") AND ("Adverse Effects"[Mesh] OR "Safety"[Mesh] OR "side effect" OR "adverse event"))	('child'/exp OR 'adolescent'/exp OR pediatric*:ti,ab OR child*:ti,ab OR adolescent*:ti,ab)<br>AND<br>('acute pain'/exp OR 'severe pain':ti,ab OR 'emergency department'/exp OR 'emergency room':ti,ab OR 'pronto soccorso':ti,ab)<br>AND<br>('opioid analgesic agent'/exp OR opioid*:ti,ab OR fentanyl:ti,ab OR morphine:ti,ab)<br>AND<br>(('intranasal administration'/exp OR 'intranasal':ti,ab OR 'nasal spray':ti,ab)<br>AND<br>('intravenous administration'/exp OR 'intravenous':ti,ab OR 'IV':ti,ab))<br>AND<br>('onset of action'/exp OR 'time to onset':ti,ab OR 'analgesic onset':ti,ab)<br>AND<br>('pain measurement'/exp OR 'pain scale':ti,ab OR 'analgesic efficacy':ti,ab)<br>AND<br>('patient acceptance'/exp OR acceptability:ti,ab OR comfort:ti,ab OR 'ease of use':ti,ab)<br>AND<br>('adverse drug reaction'/exp OR 'safety'/exp OR 'side effect':ti,ab OR 'adverse event':ti,ab)
6	(("Child"[Mesh] OR "Adolescent"[Mesh] OR "Pediatrics"[Mesh] OR child* OR pediatric* OR adolescent*)<br>AND<br>("Pain, Procedural"[Mesh] OR "Minor Surgical Procedures"[Mesh] OR "venipuncture"[Mesh] OR "wound closure"[Mesh] OR "minor procedures")<br>AND	('child'/exp OR 'adolescent'/exp OR pediatric*:ti,ab OR child*:ti,ab OR adolescent*:ti,ab)<br>AND<br>('procedural pain'/exp OR 'minor surgical procedure'/exp OR 'venipuncture'/exp OR 'wound closure'/exp OR 'minor procedure':ti,ab)<br>AND<br>('inhalation analgesia'/exp OR 'inhalation analgesia':ti,ab OR

	("Analgesia, Inhalation"[Mesh] OR "inhalation analgesia" OR "inhaled analgesia" OR "fentanyl" OR "Anesthetics, Topical"[Mesh] OR "topical anesthetic" OR "lidocaine" OR "prilocaine" OR "EMLA")<br>AND<br>("Onset of Action"[Mesh] OR "time to onset" OR "rapid analgesia" OR "fast onset" OR “efficacy” OR “effectiveness”)<br>AND<br>("Pain Measurement"[Mesh] OR "pain scale" OR "analgesic efficacy" OR<br>"Patient Acceptance of Health Care"[Mesh] OR "acceptability" OR "comfort" OR "ease of use"))	'inhaled analgesia':ti,ab OR 'fentanyl':ti,ab)<br>AND<br>('topical anesthetic'/exp OR 'lidocaine'/exp OR 'prilocaine'/exp OR 'emla':ti,ab)<br>AND<br>('onset of action'/exp OR 'time to onset':ti,ab OR 'rapid analgesia':ti,ab OR 'fast onset':ti,ab)<br>AND<br>('pain measurement'/exp OR 'pain scale'/exp OR 'analgesic efficacy':ti,ab)<br>AND<br>('patient acceptance'/exp OR acceptability:ti,ab OR comfort:ti,ab OR 'ease of use':ti,ab)
7	(("Child"[Mesh] OR "Adolescent"[Mesh] OR "Pediatrics"[Mesh] OR child* OR pediatric* OR adolescent*)<br>AND<br>("Pain, Procedural"[Mesh] OR "Minor Surgical Procedures"[Mesh] OR "venipuncture"[Mesh] OR "wound closure"[Mesh] OR "minor procedure")<br>AND<br>(("Distraction Techniques"[Mesh] OR "Virtual Reality"[Mesh] OR "Cognitive Behavioral Therapy"[Mesh] OR "Music Therapy"[Mesh] OR "Nonpharmacologic Therapies"[Mesh] OR "sucrose"[Mesh] OR "distraction" OR "virtual reality" OR "cognitive behavioral therapy" OR "music therapy" OR "sucrose" OR "non-pharmacological" OR "non pharmacological" OR "nonpharmacologic"))<br>AND<br>(("Analgesics, Oral"[Mesh] OR "Anesthetics, Topical"[Mesh] OR "pharmacologic analgesia" OR "oral analgesics" OR "topical analgesics" OR "Pain Measurement"[Mesh] OR "pain scale" OR "pain assessment")	('child'/exp OR 'adolescent'/exp OR pediatric*:ti,ab OR child*:ti,ab OR adolescent*:ti,ab)<br>AND<br>('procedural pain'/exp OR 'minor surgical procedure'/exp OR 'venipuncture'/exp OR 'wound closure'/exp OR 'minor procedure':ti,ab)<br>AND<br>(('distraction technique'/exp OR 'virtual reality'/exp OR 'cognitive behavioral therapy'/exp OR 'music therapy'/exp OR 'non pharmacological treatment'/exp OR 'sucrose'/exp)<br>OR<br>('distraction':ti,ab OR 'virtual reality':ti,ab OR 'cognitive behavioral therapy':ti,ab OR 'music therapy':ti,ab OR 'sucrose':ti,ab OR 'non-pharmacological':ti,ab OR 'non pharmacological':ti,ab OR 'nonpharmacologic':ti,ab))<br>AND<br>(('oral analgesic agent'/exp OR 'topical anesthetic'/exp OR 'pharmacologic analgesia':ti,ab OR 'oral analgesics':ti,ab OR 'topical analgesics':ti,ab))<br>AND

	AND<br>("Anxiety"[Mesh] OR "Psychomotor Agitation"[Mesh] OR "anxiety" OR "agitation" OR "psychomotor agitation")<br>AND<br>("Treatment Outcome"[Mesh] OR "Adverse Effects"[Mesh] OR "side effect" OR "adverse event"))	('pain measurement'/exp OR 'pain scale'/exp OR 'pain assessment':ti,ab)<br>AND<br>('anxiety'/exp OR 'psychomotor agitation'/exp OR 'anxiety':ti,ab OR 'agitation':ti,ab OR 'psychomotor agitation':ti,ab)<br>AND<br>('treatment outcome'/exp OR 'adverse drug reaction'/exp OR 'side effect':ti,ab OR 'adverse event':ti,ab)

Supplementary Material S2. Risk of bias assessment

Quality appraisal of original studies included into the present analysis according to the Newcastle Ottawa Scale (NOS).

	YEAR	Selection 1	Selection 2	Selection 3	Selection 4	Comparability A	Comparability B	Outcome 1	Outcome 2	Outcome 3	SCORE
PICO 1											
Abu Saad et al.	1994	+1	+1	+1	+1	0	0	+1	+1	+1	7
Bailey et al.	2010	+1	+1	+1	+1	0	0	0	+1	+1	6
Bringuier et al.	2009	+1	+1	+1	+1	+1	+1	0	+1	+1	8
PICO 2											
Baygin et al.	2011	+1	+1	+1	+1	+1	0	+1	+1	+1	8
PICO 7											
Li et al.	2016	+1	+1	+1	+1	+1	+1	+1	+1	+1	9

Appraisal of the risk of bias of randomized controlled trials included into the analyses according to the National Toxicology Program (NTP)'s Office of Health Assessment and Translation (OHAT) handbook and respective risk of bias (ROB) tool. Note: D1: possibility of selection bias; D2: exposure assessment; D3: outcome assessment; D4: confounding factors; D5: reporting bias; D6: other bias; ☹☹: definitively high; ☹: probably high; ☺: probably low; ☺☺: definitively low.

	D1	D2	D3	D4	D5	D6
PICO 2						
Salmassian et al. 2009	☺	☺	☺	☺	☺☺	☺
Abou el Fadil et al. 2019	☺	☹	☹	☺	☺☺	☺
Gazal et al. 2007	☺	☹	☹	☺	☺☺	☺
PICO 4						
Le May et al. 2017	☺☺	☺☺	☹	☺☺	☺☺	☺☺
Lu et al. 2025	☺☺	☺☺	☹	☺☺	☺☺	☺☺
PICO 5						
Fenster et al. 2018	☺	☺☺	☺	☺☺	☺☺	☺☺
PICO 7						
Liu et al. 2021	☺	☹	☹	☺	☹	☹
Ali et al. 2020	☺	☺	☺	☺	☹	☹

Supplementary Material S3. GRADE Analyses for each PICO question.

GRADE - PICO 1

Outcome	Estimate with unstructured clinical assessment	Estimate with validated pain assessment scales	Effect estimate	No. of studies	Certainty (GRADE)	Comments
**O1 – Accuracy and reliability of pain assessment**	Not estimable. No study directly evaluated the diagnostic accuracy, agreement, or reliability of unstructured clinical assessment.	Bailey et al. demonstrated good psychometric properties (convergent validity, responsiveness, and reliability) of the Verbal Numeric Rating Scale (vNRS) in children aged 6–17 years, with lower performance in children aged 4–5 years. Bringuier et al. reported good internal validity for all behavioral scales, with FLACC showing the highest sensitivity and specificity among the evaluated instruments.	Not estimable. Validated pain scales demonstrated satisfactory psychometric performance, but no direct comparison with unstructured clinical assessment was available.	2 observational validation studies	⊕⊕◯◯ **Low**	Evidence was downgraded for **indirectness**, as studies compared validated scales with other validated instruments rather than with unstructured clinical assessment. Additional heterogeneity in age groups, clinical settings, pain conditions, and assessment tools further reduced certainty.
**O2 – Adequacy of analgesic management**	Not estimable. No study included a comparison group managed exclusively through unstructured clinical assessment.	Abu-Saad et al. reported significant reductions in pain scores following analgesic administration, demonstrating responsiveness of the assessment tool. Bailey et al. similarly confirmed the responsiveness of the vNRS after analgesic treatment.	Not estimable. Available studies demonstrated responsiveness of validated scales but did not directly evaluate whether their implementation improved the appropriateness of analgesic prescribing or pain management.	2 observational studies	⊕◯◯◯ **Very low**	Evidence was downgraded for **risk of bias**, **indirectness**, and **imprecision**. No direct comparison between structured and unstructured assessment was available, and no study evaluated clinically relevant outcomes such as appropriateness of analgesic

Outcome	Estimate with unstructured clinical assessment	Estimate with validated pain assessment scales	Effect estimate	No. of studies	Certainty (GRADE)	Comments
						prescription or patient management.

GRADE - PICO 2

Outcome	Pain with paracetamol	Pain with ibuprofen	Relative Effect/Difference	N (studies)	Certainty (GRADE)	Comment
**O1 - Pain reduction (1-6 h)**	Significantly lower than placebo	Similar reduction compared to placebo, comparable to paracetamol in most RCTs.	A combined MD/RR cannot be estimated. Overall, **there is no consistent clinical difference** between acetaminophen and ibuprofen in terms of relief in the first few hours.	4 identified studies, RCTs, all congruent with O1	⊕⊕⊕◯ **Moderate**	Randomized trials, often with good description; downgrade 1 for incomplete reporting (absence of complete data for meta-analysis) and some uncertainty about blindness/allocation. Direction: both effective vs placebo; small or inconsistent differences between them.

Outcome	Placebo	Paracetamol	Ibuprofen	Combination	Effect	N (studies)	Certainty (GRADE)	Comment
**O1b – Need for morphine in the recovery room**	50% requires morphine	53%	43%	20%	Combination reduces the need for morphine from about 50% to about 20% (p=0.02). Difference between paracetamol and ibuprofen less clear (53% vs 43%).	Of the 4 studies identified, 1 RCT congruent with O1b	⊕⊕◯◯ **Low**	Downgrade due to inaccuracy (a single study with small numbers, intervals not reported) and some risk of bias (not all randomization and blindness procedures are explained in detail in the file).
**O1c – Need for codeine at home**	50%	50%	37%	13%	Combination reduces the need from ~50% to 13% (p=0.003). Ibuprofen alone 37% (intermediate).	Of the 4 studies identified, 1 RCT congruent with O1c	⊕⊕◯◯ **Low**	Suggestive evidence but limited to a single study.<br><br>It appears that ibuprofen alone requires less codeine than acetaminophen, but the data are not sufficient for robust conclusions.

Outcome	Paracetamol	Ibuprofen	Relative effect	N (studies)	Certainty (GRADE)	Comment
**O2 – Adverse events (GI, renal, others)**	Rare events, not clearly greater than placebo; partial reporting.	Like paracetamol, no clear signs of excess; partial reporting.	Not restimable	Of the 4 studies, all RCTs, all compatible with O2, but the concept of safety is a secondary outcome, with an incomplete description	⊕◯◯◯ **Very low**	Downgrade due to risk of bias (safety not systematically measured), indirectness (short follow-up, selected population), and strong inaccuracy (few events, small numbers).<br>Safety differences cannot be ruled out, especially for rare events.

GRADE - PICO 7

Outcome	Standard management	Non-pharmacological techniques	Effect	N (studies)	Certainty (GRADE)	Comment
**O1 – Pain reduction (FPS-R and other scales)**	Liu 2021: Median FPS-R 4 (IQR 2–6) during IV insertion.	Median FPS-R 2 (IQR 0–4); **p = 0.13** (not significant).	Trend toward lower scores with robot distractions, but no evidence of significant difference; no studies directly compare non-drug vs. drug alone.	3 studies, of which 2 congruent with O1, in particular 1 RCT with numerical data on pain (Liu 2021) + 1 observational study	⊕◯◯◯ **Very low**	Downgrade for: (1) **risk of design bias/limitations** (only one small RCT), (2) **inaccuracy** (CIs not reported; p=0.13), (3) **marked indirectness** (comparison is not non-drug vs. drug isolates).

Outcome	Without structured intervention	With non-pharmacological intervention	Effect	N (studies)	Certainty (GRADE)	Comment
**O2a – Negative Emotional Behaviors (CEMS)**	Li 2016: Higher CEMS scores, indicative of more negative emotions.	Li 2016: significantly lower scores;<br><br>t(180) = –7.3 (3–7 years)<br><br>t(120) = –8.1 (8–12 years); **p < 0.001**.	Wide and very significant differences in favor of hospital gaming; No comparative data with "drug only", however.	3 studies, of which only 1 congruent with O2a, of quasi-experimental design (Li 2016)	⊕⊕◯◯ **Low**	Downgrade 1 for non-randomized design (possible confounding), downgrade 1 for **indirectness** (does not compare directly vs pharmacotherapy). No downgrade due to inconsistency (very consistent and robust effect in the single study).
**O2b – Parenting Anxiety (STAI-S)**	Liu 2021: After procedure, STAI-S 36.7 ± 11.9.	STAI-S post-procedure 31.3 ± 8.5; **p = 0.04**.	Statistically significant reduction in parental anxiety.	3 studies, including only 1 RCT congruous with O2b	⊕⊕◯◯ **Low**	Downgrade 1 for limited sample size/inaccuracy, 1 for **indirectness**: the outcome concerns the parents, not

Outcome	Without structured intervention	With non-pharmacological intervention	Effect	N (studies)	Certainty (GRADE)	Comment
						directly the child's pain, and does not compare vs "drug only".

Outcome	Standard	Non-pharmacological	Effect	Certainty
**O3 – Adverse events**	Not reported in a structured way.	3 studies congruent with the definition of O3, but the data is not reported in a structured way; no obvious signs of damage.	Not restimable	⊕◯◯◯ **Very low**

Supplementary Material S4: PRISMA checklist 2020.

Section and Topic	Item #	Checklist item	Location where item is reported
**TITLE**			
Title	1	Identify the report as a systematic review.	1
**ABSTRACT**			
Abstract	2	See the PRISMA 2020 for Abstracts checklist.	2
**INTRODUCTION**			
Rationale	3	Describe the rationale for the review in the context of existing knowledge.	3
Objectives	4	Provide an explicit statement of the objective(s) or question(s) the review addresses.	3
**METHODS**			
Eligibility criteria	5	Specify the inclusion and exclusion criteria for the review and how studies were grouped for the syntheses.	3-4
Information sources	6	Specify all databases, registers, websites, organisations, reference lists and other sources searched or consulted to identify studies. Specify the date when each source was last searched or consulted.	4-5
Search strategy	7	Present the full search strategies for all databases, registers and websites, including any filters and limits used.	4-5
Selection process	8	Specify the methods used to decide whether a study met the inclusion criteria of the review, including how many reviewers screened each record and each report retrieved, whether they worked independently, and if applicable, details of automation tools used in the process.	4-5
Data collection process	9	Specify the methods used to collect data from reports, including how many reviewers collected data from each report, whether they worked independently, any processes for obtaining or confirming data from study investigators, and if applicable, details of automation tools used in the process.	4-5
Data items	10a	List and define all outcomes for which data were sought. Specify whether all results that were compatible with each outcome domain in each study were sought (e.g. for all measures, time points, analyses), and if not, the methods used to decide which results to collect.	4-5
	10b	List and define all other variables for which data were sought (e.g. participant and intervention characteristics, funding sources). Describe any assumptions made about any missing or unclear information.	4-5
Study risk of bias assessment	11	Specify the methods used to assess risk of bias in the included studies, including details of the tool(s) used, how many reviewers assessed each study and whether they worked independently, and if applicable, details of automation tools used in the process.	4-5
Effect measures	12	Specify for each outcome the effect measure(s) (e.g. risk ratio, mean difference) used in the synthesis or presentation of results.	4-5
Synthesis methods	13a	Describe the processes used to decide which studies were eligible for each synthesis (e.g. tabulating the study intervention characteristics and comparing against the planned groups for each synthesis (item #5)).	4-5
	13b	Describe any methods required to prepare the data for presentation or synthesis, such as handling of missing summary statistics, or data	4-5

Section and Topic	Item #	Checklist item	Location where item is reported
		conversions.	
	13c	Describe any methods used to tabulate or visually display results of individual studies and syntheses.	3-5
	13d	Describe any methods used to synthesize results and provide a rationale for the choice(s). If meta-analysis was performed, describe the model(s), method(s) to identify the presence and extent of statistical heterogeneity, and software package(s) used.	3-5
	13e	Describe any methods used to explore possible causes of heterogeneity among study results (e.g. subgroup analysis, meta-regression).	3-5
	13f	Describe any sensitivity analyses conducted to assess robustness of the synthesized results.	3-5
Reporting bias assessment	14	Describe any methods used to assess risk of bias due to missing results in a synthesis (arising from reporting biases).	3-5
Certainty assessment	15	Describe any methods used to assess certainty (or confidence) in the body of evidence for an outcome.	3-5
**RESULTS**			
Study selection	16a	Describe the results of the search and selection process, from the number of records identified in the search to the number of studies included in the review, ideally using a flow diagram.	5-21
	16b	Cite studies that might appear to meet the inclusion criteria, but which were excluded, and explain why they were excluded.	5-21
Study characteristics	17	Cite each included study and present its characteristics.	5-21
Risk of bias in studies	18	Present assessments of risk of bias for each included study.	5-21
Results of individual studies	19	For all outcomes, present, for each study: (a) summary statistics for each group (where appropriate) and (b) an effect estimate and its precision (e.g. confidence/credible interval), ideally using structured tables or plots.	5-21
Results of syntheses	20a	For each synthesis, briefly summarise the characteristics and risk of bias among contributing studies.	5-21
	20b	Present results of all statistical syntheses conducted. If meta-analysis was done, present for each the summary estimate and its precision (e.g. confidence/credible interval) and measures of statistical heterogeneity. If comparing groups, describe the direction of the effect.	5-21
	20c	Present results of all investigations of possible causes of heterogeneity among study results.	5-21
	20d	Present results of all sensitivity analyses conducted to assess the robustness of the synthesized results.	5-21
Reporting biases	21	Present assessments of risk of bias due to missing results (arising from reporting biases) for each synthesis assessed.	5-21
Certainty of evidence	22	Present assessments of certainty (or confidence) in the body of evidence for each outcome assessed.	5-21
**DISCUSSION**			
Discussion	23a	Provide a general interpretation of the results in the context of other evidence.	21-24
	23b	Discuss any limitations of the evidence included in the review.	23

Section and Topic	Item #	Checklist item	Location where item is reported
	23c	Discuss any limitations of the review processes used.	23
	23d	Discuss implications of the results for practice, policy, and future research.	24
**OTHER INFORMATION**			
Registration and protocol	24a	Provide registration information for the review, including register name and registration number, or state that the review was not registered.	24
	24b	Indicate where the review protocol can be accessed, or state that a protocol was not prepared.	24
	24c	Describe and explain any amendments to information provided at registration or in the protocol.	24
Support	25	Describe sources of financial or non-financial support for the review, and the role of the funders or sponsors in the review.	24
Competing interests	26	Declare any competing interests of review authors.	24
Availability of data, code and other materials	27	Report which of the following are publicly available and where they can be found: template data collection forms; data extracted from included studies; data used for all analyses; analytic code; any other materials used in the review.	24

From: Page MJ, McKenzie JE, Bossuyt PM, Boutron I, Hoffmann TC, Mulrow CD, et al. The PRISMA 2020 statement: an updated guideline for reporting systematic reviews. BMJ 2021;372:n71. doi: 10.1136/bmj.n71. This work is licensed under CC BY 4.0. To view a copy of this license, visit https://creativecommons.org/licenses/by/4.0/
